# Comparison of Morphologic Parameters of Temporomandibular Joint for Asymptomatic Subjects Using the Two-Dimensional and Three-Dimensional Measuring Methods

**DOI:** 10.1155/2017/5680708

**Published:** 2017-05-02

**Authors:** Yuanli Zhang, Xianchao Xu, Zhan Liu

**Affiliations:** Provincial Key Lab for Biomechanical Engineering, Sichuan University, Chengdu 610065, China

## Abstract

The differences of temporomandibular joint (TMJ) morphologic parameters by using two-dimensional (2D) and three-dimensional (3D) measuring methods were compared. Ten asymptomatic subjects (26.75 ± 4.89 years) were randomly recruited. The 3D models of the maxilla, mandible, and teeth were reconstructed according to cone-beam computed tomography (CBCT) image data. The morphologic parameters of TMJs were measured by the 2D CBCT measuring method (group A) and the 3D reconstruction model measuring method (group B), respectively. The morphologic parameters in each group were assessed by paired samples *t*-test, and the statistical significance was achieved when *p* < 0.05. The horizontal condylar angle (HCA), sagittal ramus angle (SRA), medial joint space (MJS), lateral joint space (LJS), superior joint space (SJS), and anterior joint space (AJS) in group A were significantly smaller than those in group B (*p* < 0.05). The HCA on the left side was significantly smaller than that on the right side in group A (*p* < 0.05). However, all the morphologic parameters in group B were not significantly different between left and right sides. In conclusion, there were significant differences for the morphologic parameters of TMJ measured on 2D CBCT and 3D models. 3D measuring method should be used for the detection of TMJ morphology in clinical practice.

## 1. Introduction

Temporomandibular joints (TMJs) are a pair of complex and highly mobile joints. TMJs are the most active joints in the human body with more than 2000 movements each day during chewing, biting, swallowing, talking, and snoring [1]. Dupuy-Bonafe et al. indicated that the morphologic examination of the TMJ has important applications in the domain of TMJ pathology [2]. Therefore, the reasonable measurement of TMJ morphologic parameters will help us to better understand the structure and function of TMJ.

Due to the complexity of the skull base and TMJ components, many studies have investigated the TMJ morphologic parameters using different types of imaging techniques. Conventional X-rays were first used to assess the morphology of mandibular condyle and articular eminence [3]. Subsequently, the lateral cephalogram was used to determine the selection criteria for ordering a corrected lateral tomogram of the TMJ [4], to investigate the possible association between the joint structure and condylar position and craniofacial morphology [5], and to measure the TMJ positions of Malays and Chinese with facial asymmetry for assessing its impact on the temporomandibular disorders [6]. After that, computed tomography (CT) images were widely used for the morphologic detection of TMJ [2, 7, 8]. In recent years, the micro-CT, panoramic radiography (PR), cone-beam computed tomography (CBCT), and magnetic resonance imaging (MRI) were used for research of TMJ morphology [9–14]. In Zhao et al.'s study, micro-CT images were used to observe morphologic changes of the TMJ condyle under mandibular deviation continuously in an animal model [9]. PR images were used to diagnose the condylar bone defects in 5 dried human skulls [10]. CBCT images were used to analyze the facial morphologic characteristics of female patients with skeletal class II deformity [11] and to examine the relationship between the thickness of the glenoid fossa roof and condyle morphology [12]. MRI images were used to evaluate the change of morphologic symmetry of TMJ during natural course of teen-age unilateral anterior disc displacement [13]. Meanwhile, the three-dimensional (3D) models were also used to investigate the condylar morphology and TMJ osteoarthritis [15–17].

Most of the related studies have been limited to two-dimensional (2D) measuring method to assess the TMJ morphologic parameters. However, these results measured by 2D method do not show the spatial position of the structures in TMJ. The 3D measuring method was used to assess the morphology of condyle, but other TMJ structures, such as glenoid fossa and articular eminence, were not included in the previous studies. Furthermore, the comparisons between the TMJ morphologic parameters measured by 2D and 3D methods have not been studied. The aim of this study was to compare the differences of TMJ morphologic parameters using 2D CBCT and 3D model measuring methods. This work has been presented as a poster at the ORS 2017 Annual Meeting, and it was considered as a valuable research by the experts.

## 2. Materials and Methods

### 2.1. Subjects and Data Acquisition

This study consisted of 10 asymptomatic subjects (4 females and 6 males, 26.75 ± 4.89 years old). The subjects were randomly identified and recruited by a dentist in the Affiliated Hospital of Stomatology, Chongqing Medical University from January 2015 to January 2016. This study was approved by the Affiliated Hospital of Stomatology of Chongqing Medical University Institutional Review Board, and all participants signed an informed consent agreement. The inclusion criteria of asymptomatic subjects were as follows: healthy physical condition, no TMJ disorder symptoms, no degenerative joint disease, and facial symmetry with no prior TMJ-related procedures.

CBCT data for all subjects were collected at the Affiliated Hospital of Stomatology, Chongqing Medical University. The maxilla and mandible were scanned using a CBCT machine (KaVo 3D eXam, Germany) with a complete head view. All images were taken following a standardized protocol for patient positioning and exposure parameter setting (120 kVp, 3–8 mA, 20 sec, and 0.4 mm voxel resolution). The resolution of cross-sectional images was 400 pixels × 400 pixels, and the pixel size was 0.4 mm. The CBCT scans consisted of a total amount of 290 to 330 images with the slice thicknesses of 0.4 mm. The CBCT images were reformatted into Digital Imaging and Communications in Medicine (DICOM) format.

### 2.2. 3D Modeling

**Figure 1:**
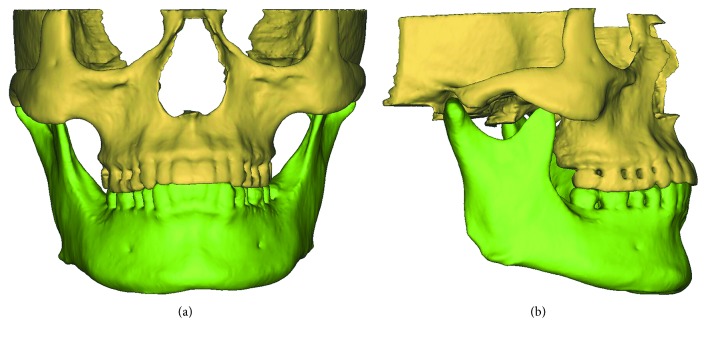
The 3D model of an asymptomatic subject: (a) front view and (b) lateral view.

According to the Hounsfield units (HU), the cortical bone, cancellous bone, and teeth were separated in MIMICS (Materialise, Leuven, Belgium), respectively. The boundaries of the regions of the maxilla, mandible, and teeth were accurately distinguished on each slice of CBCT images. Subsequently, the 3D models of the maxilla, mandible, and teeth were reconstructed in MIMICS for the 10 asymptomatic subjects (Figure 1).

### 2.3. TMJ Morphologic Analysis

Based on previous research [18, 19], the horizontal condylar angle (HCA), coronal condylar angle (CCA), sagittal ramus angle (SRA), coronal condylar width (CCW), medial joint space (MJS), lateral joint space (LJS), superior joint space (SJS), anterior joint space (AJS), and posterior joint space (PJS) were selected to investigate the morphology of TMJ. The above nine morphologic parameters were measured in the 2D CBCT images and 3D models of all the subjects, respectively.

**Figure 2:**
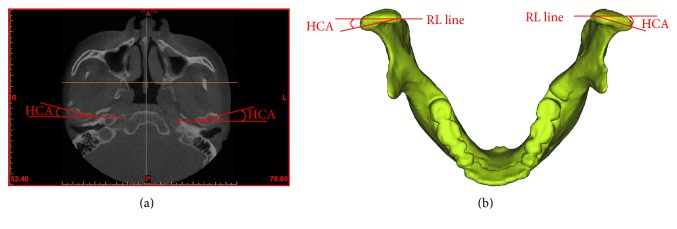
Measurements of the HCA in the horizontal view: (a) on the CBCT images and (b) in the 3D models. HCA: horizontal condylar angle.

The HCA was measured in the horizontal view (paralleling to the Frankfurt horizontal (FH) plane):

1. HCA: the angle between the condylar long axis (the line between the most medial and lateral points) and the RL line (the line between the most anterior points of the bilateral auricles) [19], as shown in Figure 2

**Figure 3:**
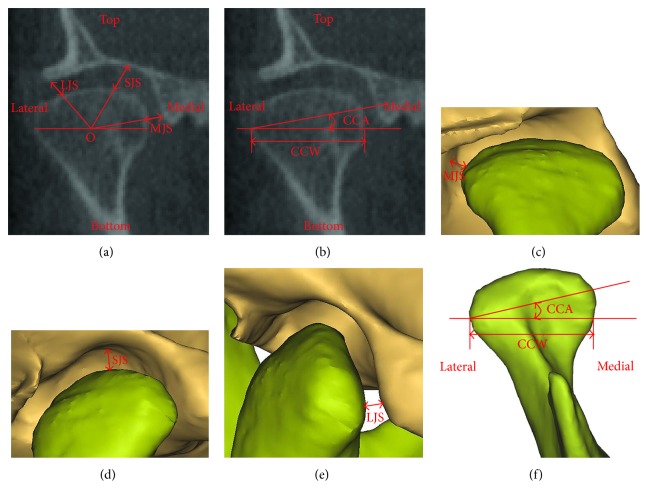
Measurements of the MJS, SJS, LJS, CCA, and CCW in the coronal view: (a and b) on the CBCT images and (c, d, e, and f) in the 3D models. The point O is the midpoint of CCW in part (a). CCA: coronal condylar angle; CCW: coronal condylar width; MJS: medial joint space; LJS: lateral joint space; SJS: superior joint space.

The CCA, CCW, MJS, SJS, and LJS were measured in the coronal view (Figure 3).

(2) CCA: the angle between the FH plane and the condylar long axis
(3) CCW: the length of a line segment paralleled to the FH plane and passed through the most lateral point of the condyle
(4) MJS: the distance between the most medial point of the condyle and the articular fossa
(5) SJS: the distance between the most superior point of the condyle and the articular fossa
(6) LJS: the distance between the most lateral point of the condyle and the articular fossa

**Figure 4:**
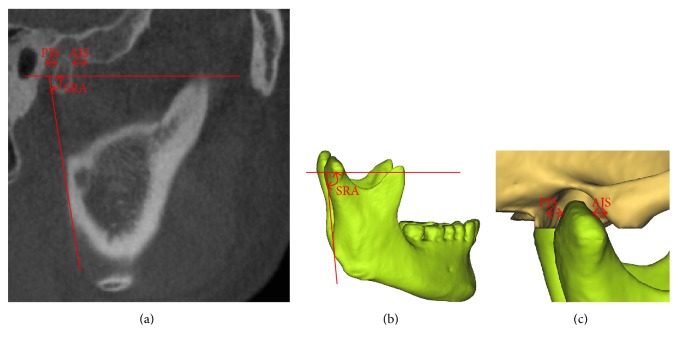
Measurements of the SRA, PJS, and AJS in the sagittal view: (a) on the CBCT images and (b and c) in the 3D models. SRA: sagittal ramus angle; AJS: anterior joint space; PJS: posterior joint space.

The SRA, PJS, and AJS were measured in the sagittal view (Figure 4).

(7) SRA: the angle between the FH plane and the tangential line to the posterior outline of mandibular ramus
(8) PJS: the distance paralleled to the FH plane between the posterior points of the condyle and the articular eminence outline
(9) AJS: the distance paralleled to the FH plane between the anterior points of the condyle and the articular fossa outline.

In this study, the nine morphologic parameters for each model were re-evaluated thrice within a one-week interval by three authors of this study using 2D and 3D measuring methods, respectively. The correlation coefficients of the results in each measurement were greater than 0.95. Therefore, the repeatability of the measurements was acceptable in this study.

### 2.4. Statistical Analysis

The morphologic differences of TMJ between 2D and 3D measuring methods were analyzed using paired samples *t*-test. In addition, the comparisons were performed in the following: (1) between the 2D measuring results (group A) and 3D measuring results (group B) of the nine morphologic parameters and (2) between the left TMJ and right TMJ in groups A and B, respectively. SPSS 20.0 (SPSS Inc., Chicago, IL) was used for the analysis. The statistical significance was achieved when *p* < 0.05.

## 3. Results

The angles (HCA and SRA) and the joint spaces (MJS, LJS, SJS, and AJS) were found to be statistically significant between the groups A and B (Table 1). The HCA in group B was significantly larger than that in group A (*p* < 0.05). However, there was no significant difference for CCA between groups A and B (*p* > 0.05). The SRA in group B was significantly higher than that in group A (*p* < 0.05). Meanwhile, the MJS, LJS, and SJS in group B were highly significantly greater than those in group A (*p* < 0.01). The AJS in group B was also significantly greater than that in group A (*p* < 0.05). Although there were no significant differences for CCW and PJS between the groups A and B, the mean values of CCW and PJS in group B were still larger than those in group A (Table 1).

The morphologic differences of TMJ between the left and right sides were compared in group A and group B, respectively. The results showed that the HCA on the left side was significantly smaller than that on the right side in group A (*p* < 0.05). There was no significant difference for other morphologic parameters between the left TMJ and right TMJ in group A (*p* > 0.05). However, there was no significant difference for all the nine morphologic parameters between the left and right sides in group B (Table 2).

## 4. Discussion

The morphologic parameters of TMJ obtained from the medical images were the projection of the actual morphology of TMJ in a certain plane, such as horizontal plane, coronal plane, and sagittal plane. Therefore, these morphologic parameters measured by the medical images (2D method) did not show the spatial position relation of the structures in the TMJs. At present, a few stomatology hospitals are trying to establish the 3D measurement standard of the morphologic parameters of TMJ. However, the differences of the morphologic parameters of TMJ between the 2D and 3D measuring methods are still unclear. In this study, the TMJ morphology was evaluated by 2D and 3D measuring methods in asymptomatic subjects. The results indicated that there were significant differences between the two methods.

The joint space (MJS, LJS, SJS, AJS, and PJS) of TMJ would determine the disc position in the TMJ [20]. The condylar angle (HCA and CCA) could be an aetiological factor for disc displacement and degenerative joint disease [19–21]. The ramus angle (SRA) would determine the symmetry of mandible [19, 20]. Meanwhile, the AJS, PJS, MJS, LJS, SJS, and CCW were the frequently used parameters in clinics to examine the morphology of TMJ. Therefore, the above nine morphologic parameters were used to investigate the TMJ morphology in this study. The 2D CBCT measuring method has been validated in the related studies [18, 19, 22–24]. The 3D measuring method also has been used to evaluate the condylar morphology [15, 17, 25]. The mean values of CCA were 11.9° and 12.7° on the left and right sides measured in 2D CBCT from Ueki et al.'s study [19]. In this study, the mean angles of CCA were 12.48 ± 0.55° and 12.73 ± 0.51° on the left and right sides, respectively. The mean distances of SJS and PJS were 1.8 mm and 2.5 mm measured in 2D CBCT, respectively, from Ueki et al. [19]. In this study, the mean distances of SJS and PJS were 2.05 ± 0.12 mm and 2.53 ± 0.47 mm, respectively. The mean value of CCW was 18.21 ± 2.23 mm measured in 2D CBCT in this study, close to Al-koshab et al.'s results of 17.80 mm [26]. In Martins et al.'s study [27], the mean values of MJS and SJS for asymptomatic subjects were 2.94 mm and 2.55 mm measured in 3D models, respectively. In this study, the mean values of MJS and SJS were 2.81 ± 0.35 mm and 2.27 ± 0.18 mm measured in 3D models, respectively. Other morphologic parameters of TMJ measured in this study were also similar to previous studies [18, 28–30]. Therefore, the results were accurate in this study.

The transverse dimension of condyloid process was 19.5 ± 2.4 mm measured in the Chinese skull specimens [31]. In this study, the CCW was 18.54 ± 2.12 mm and the CCA was 12.93 ± 0.59° in 3D models, so the transverse dimension of condyloid process was 19.11 mm in 3D models, and it was 98.00% of the actual value. However, the CCW was 18.21 ± 2.23 mm and the CCA was 12.61 ± 0.39° in 2D CBCT, so the transverse dimension of condyloid process was 18.58 mm in 2D CBCT, and it was 95.28% of the actual value. Meanwhile, the angle of the long axis of bilateral condyle was 145°~160° measured in the Chinese skull specimens [32], that is to say, the average HCA was 13.75°. In this study, the HCA was 12.72 ± 0.23° in 3D models, and it was 92.51% of the actual value. However, the HCA was 12.19 ± 0.57° in 2D CBCT, and it was 88.65% of the actual value. Therefore, the morphologic parameters of TMJ in 3D models were more accurate than those in the 2D CBCT, and then the morphologic parameters of TMJ measured in 3D models could represent the actual morphology of TMJ to some extent.

The results in Table 1 showed that the nine morphologic parameters of TMJ in group A were smaller than those in group B. The results suggested that the angles and distances measured by 2D method were smaller than those measured by 3D method. This finding also indicated that the 2D method may underestimate the size of TMJ morphologic parameters. These smaller results measured by 2D method may be caused by the projective values that were less than the actual values. The degree of motion of the cervical spine during CT imaging could also affect the projection of the TMJ morphologic parameters in the 2D image. In addition, CBCT images can only show the morphology and position of TMJ in horizontal, coronal, and sagittal planes. Clinicians need to combine the 2D images and their clinic experience to diagnose the TMJs. However, 3D reconstructed models can accurately provide the stereoscopic structure and spatial anatomical location of TMJ, which can better help clinicians to diagnose the morphology of TMJs along with the symptom and sign. The HCA, SRA, MJS, LJS, SJS, and AJS in group A were significantly smaller than those in group B, consistent with Zhang et al.'s study [18]. In particular, the values of MJS, LJS, and SJS measured in 2D CBCT were 81.85%, 84.82%, and 90.31% of those measured in 3D models, respectively. The significant difference in 2D image measurement could lead to the misdiagnosis of TMJ. The CCA, CCW, and PJS measured by 2D method were also a little smaller than those measured by 3D method. Due to the small projective angles, the projective values of CCA, CCW, and PJS were close to their actual values. In summary, there were significant differences in the results of the morphologic parameters of TMJ between 2D and 3D measurements. In addition, 3D reconstructed model can show the stereoscopic structure and spatial position of TMJ and would better help clinicians to accurately diagnose the morphology of TMJ, in accordance with Cevidanes and Al-Saleh et al.'s studies [15–17].

There was significant difference for HCA between the left and right sides in group A. However, all the subjects in this study were asymptomatic and symmetric, in accordance with the 3D measured results in this study. This finding suggested that the HCA measured in 2D CBCT was not consistent with the actual situation. The asymmetric parameter of TMJ measured in 2D image may be caused by the subjects' deflected scanning body position. The 3D reconstructed models of TMJ are not affected by the subjects' scanning body position. Therefore, some parameters of TMJ morphology measured by 2D measuring method were inaccurate to some extent. Moreover, the biased results measured in 2D image may lead to the misdiagnosis of TMJ morphology for clinicians. There were no significant differences for all the nine morphologic parameters of TMJ between the left and right sides in group B. The results were consistent with the fact of the ten asymptomatic subjects recruited in this study. Generally, we believe that the morphologic parameters measured in the 3D reconstructed models can represent the actual morphology of TMJ. Therefore, the values of TMJ morphology measured by 3D measuring method were more accurate than those measured by 2D method. 3D measuring method should be used for the detection of TMJ morphology in clinical practice.

One major limitation of the current study is that the sample size is a little small. Certainly, a multicentric study with a larger sample should be necessary to confirm our findings.

## 5. Conclusions

There were significant differences for the morphologic parameters of TMJ measured in 2D CBCT images and 3D reconstructed models. In addition, the 3D reconstructed model could show the actual stereoscopic structure and spatial position of TMJ, so the 3D measuring method is more accurate for clinicians to investigate the morphology of TMJ.

## Figures and Tables

**Table 1:** Mean and standard deviation of the morphologic parameters for the ten asymptomatic subjects measured in 2D CBCT (group A) and 3D model (group B).

Morphologic parameters	Group A (*n* = 10)	Group B (*n* = 10)	*p* value
HCA (°)	12.19 ± 0.57	12.72 ± 0.23	0.034^∗^
CCA (°)	12.61 ± 0.39	12.93 ± 0.59	0.123
SRA (°)	76.68 ± 3.54	80.37 ± 1.23	0.012^∗^
CCW (mm)	18.21 ± 2.23	18.54 ± 2.12	0.607
MJS (mm)	2.30 ± 0.20	2.81 ± 0.35	0.002^∗∗^
LJS (mm)	2.57 ± 0.19	3.03 ± 0.53	0.007^∗∗^
SJS (mm)	2.05 ± 0.12	2.27 ± 0.18	0.001^∗∗^
AJS (mm)	2.47 ± 0.24	2.77 ± 0.43	0.039^∗^
PJS (mm)	2.53 ± 0.47	2.62 ± 0.66	0.601

Note: *p* > 0.05, not significant. ^∗^Statistically significant difference between group A and group B by paired samples *t*-test (*p* < 0.05). ^∗∗^Highly statistically significant difference between group A and group B by paired samples *t*-test (*p* < 0.01). HCA: horizontal condylar angle; CCA: coronal condylar angle; SRA: sagittal ramus angle; CCW: coronal condylar width; MJS: medial joint space; LJS: lateral joint space; SJS: superior joint space; AJS: anterior joint space; PJS: posterior joint space; 2D: two-dimensional; 3D: three-dimensional.

**Table 2:** Mean and standard deviation of the morphologic parameters for the ten asymptomatic subjects on the left and right TMJs.

Morphologic parameters	Group A (*n* = 10)			Group B (*n* = 10)		
	Left TMJ	Right TMJ	*p* value	Left TMJ	Right TMJ	*p* value
HCA (°)	11.91 ± 0.55	12.46 ± 0.74	^∗^	12.57 ± 0.37	12.87 ± 0.31	NS
CCA (°)	12.48 ± 0.55	12.73 ± 0.51	NS	12.91 ± 0.63	12.95 ± 0.60	NS
SRA (°)	76.65 ± 3.28	76.70 ± 3.86	NS	80.11 ± 1.52	80.62 ± 1.30	NS
CCW (mm)	18.54 ± 2.56	17.88 ± 2.04	NS	18.69 ± 2.05	18.39 ± 2.28	NS
MJS (mm)	2.27 ± 0.28	2.32 ± 0.21	NS	2.84 ± 0.50	2.78 ± 0.46	NS
LJS (mm)	2.54 ± 0.24	2.59 ± 0.19	NS	3.09 ± 0.77	2.96 ± 0.47	NS
SJS (mm)	2.03 ± 0.14	2.07 ± 0.15	NS	2.26 ± 0.19	2.28 ± 0.18	NS
AJS (mm)	2.55 ± 0.56	2.39 ± 0.35	NS	2.64 ± 0.65	2.90 ± 0.48	NS
PJS (mm)	2.49 ± 0.44	2.57 ± 0.54	NS	2.55 ± 0.82	2.68 ± 0.61	NS

Note: NS: not significant (*p* > 0.05). ^∗^Statistically significant difference between the left and right TMJs by paired samples *t*-test (*p* < 0.05). HCA: horizontal condylar angle; CCA: coronal condylar angle; SRA: sagittal ramus angle; CCW: coronal condylar width; MJS: medial joint space; LJS: lateral joint space; SJS: superior joint space; AJS: anterior joint space; PJS: posterior joint space.

## References

[1] Mahdian N., Dostalova T., Danek J. (2013). 3D reconstruction of TMJ after resection of the cyst and the stress-strain analyses. *Computer Methods and Programs in Biomedicine*.

[2] Dupuy-Bonafe I., Otal P., Montal S., Bonafe A., Maldonado I. L. (2014). Biometry of the temporomandibular joint using computerized tomography. *Surgical and Radiologic Anatomy*.

[3] Kawashima T., Abe S., Okada M., Kawada E., Saitoh C., Ide Y. (1997). Internal structure of the temporomandibular joint and the circumferential bone: comparison between dentulous and edentulous specimens. *The Bulletin of Tokyo Dental College*.

[4] Luke L. S., Lee P., Atchison K. A., White S. C. (1997). Orthodontic residents’ indications for use of the lateral TMJ tomogram and the posteroanterior cephalogram. *Journal of Dental Education*.

[5] Kikuchi K., Takeuchi S., Tanaka E., Shibaguchi T., Tanne K. (2003). Association between condylar position, joint morphology and craniofacial morphology in orthodontic patients without temporomandibular joint disorders. *Journal of Oral Rehabilitation*.

[6] Rakesh N., Devi B. K. Y., Patil D. J., Nagi R. (2014). Assessment of cervical spine postural disorders in patients with temporomandibular dysfunction: a radiographic evaluation. *Oral Radiology*.

[7] Rehman T. A., Gibikote S., Ilango N., Thaj J., Sarawagi R., Gupta A. (2009). Bifid mandibular condyle with associated temporomandibular joint ankylosis: a computed tomography study of the patterns and morphological variations. *Dentomaxillofacial Radiology*.

[8] Minagi S., Sato T., Kishi K., Natsuaki N., Akamatsu Y. (2000). Comparative study of the temporomandibular joint space in maximum intercuspation and canine edge-to-edge positions in deep bite and non-deep bite subjects. *Journal of Oral Rehabilitation*.

[9] Zhao C., Kurita H., Kurashina K., Hosoya A., Arai Y., Nakamura H. (2010). Temporomandibular joint response to mandibular deviation in rabbits detected by 3D micro-CT imaging. *Archives of Oral Biology*.

[10] Salemi F., Shokri A., Mortazavi H., Baharvand M. (2015). Diagnosis of simulated condylar bone defects using panoramic radiography, spiral tomography and cone-beam computed tomography: a comparison study. *Journal of Clinical and Experimental Dentistry*.

[11] Chen S., Lei J., Fu K.-Y., Wang X., Yi B. (2015). Cephalometric analysis of the facial skeletal morphology of female patients exhibiting skeletal class II deformity with and without temporomandibular joint osteoarthrosis. *PloS One*.

[12] Ejima K., Schulze D., Stippig A., Matsumoto K., Rottke D., Honda K. (2013). Relationship between the thickness of the roof of glenoid fossa, condyle morphology and remaining teeth in asymptomatic European patients based on cone beam CT data sets. *Dentomaxillofacial Radiology*.

[13] Xie Q. Y., Yang C., He D. M. (2016). Will unilateral temporomandibular joint anterior disc displacement in teenagers lead to asymmetry of condyle and mandible? A longitudinal study. *Journal of Cranio-Maxillofacial Surgery*.

[14] Chien-Chih C., Cheng-Chung L., Yunn-Jy C., Tung-Wu L., Chun-Yu H. (2013). Accuracy assessment of marker-cluster registration method for measuring temporomandibular kinematics using cone-beam computed tomography with fluoroscopy. *Journal of Medical and Biological Engineering*.

[15] Cevidanes L. H. S., Walker D., Schilling J. (2014). 3D osteoarthritic changes in TMJ condylar morphology correlates with specific systemic and local biomarkers of disease. *Osteoarthritis and Cartilage*.

[16] Cevidanes L. H. S., Gomes L. R., Jung B. T. (2015). 3D superimposition and understanding temporomandibular joint arthritis. *Orthodontics & Craniofacial Research*.

[17] Al-Saleh M. A. Q., Alsufyani N., Flores-Mir C., Nebbe B., Major P. W. (2015). Changes in temporomandibular joint morphology in class II patients treated with fixed mandibular repositioning and evaluated through 3D imaging: a systematic review. *Orthodontics & Craniofacial Research*.

[18] Zhang Y. L., Song J. L., Xu X. C. (2016). Morphologic analysis of the temporomandibular joint between patients with facial asymmetry and asymptomatic subjects by 2D and 3D evaluation a preliminary study. *Medicine*.

[19] Ueki K., Moroi A., Sotobori M. (2012). Changes in temporomandibular joint and ramus after sagittal split ramus osteotomy in mandibular prognathism patients with and without asymmetry. *Journal of Cranio-Maxillofacial Surgery*.

[20] Ueki K., Nakagawa K., Takatsuka S. (2000). Temporomandibular joint morphology and disc position in skeletal class III patients. *Journal of Cranio-Maxillofacial Surgery*.

[21] Sanroman J. F., Gonzalez J. M. G., del Hoyo J. A. (1998). Relationship between condylar position, dentofacial deformity and temporomandibular joint dysfunction: an MRI and CT prospective study. *Journal of Cranio-Maxillofacial Surgery*.

[22] Cevidanes L. H. S., Hajati A. K., Paniagua B. (2010). Quantification of condylar resorption in temporomandibular joint osteoarthritis. *Oral Surgery Oral Medicine Oral Pathology Oral Radiology and Endodontology*.

[23] Ueki K., Degerliyurt K., Hashiba Y., Marukawa K., Nakagawa K., Yamamoto E. (2008). Horizontal changes in the condylar head after sagittal split ramus osteotomy with bent plate fixation. *Oral Surgery Oral Medicine Oral Pathology Oral Radiology and Endodontology*.

[24] Borahan M. O., Mayil M., Pekiner F. N. (2016). Using cone beam computed tomography to examine the prevalence of condylar bony changes in a Turkish subpopulation. *Nigerian Journal of Clinical Practice*.

[25] Xi T., van Loon B., Fudalej P., Berge S., Swennen G., Maal T. (2013). Validation of a novel semi-automated method for three-dimensional surface rendering of condyles using cone beam computed tomography data. *International Journal of Oral and Maxillofacial Surgery*.

[26] Al-koshab M., Nambiar P., John J. (2015). Assessment of condyle and glenoid fossa morphology using CBCT in South-East Asians. *PloS One*.

[27] Martins E., Silva J.-C., Pires C. A., Ponces-Ramalhao M.-J.-F., Lopes J.-D. (2015). Coronal joint spaces of the temporomandibular joint: systematic review and meta-analysis. *Journal of Clinical and Experimental Dentistry*.

[28] Gomes L. R., Gomes M., Jung B. (2015). Diagnostic index of three-dimensional osteoarthritic changes in temporomandibular joint condylar morphology. *Journal of Medical Imaging*.

[29] Chen C.-C., Lin C.-C., Lu T.-W., Chiang H., Chen Y.-J. (2013). Feasibility of differential quantification of 3D temporomandibular kinematics during various oral activities using a cone-beam computed tomography-based 3D fluoroscopic method. *Journal of Dental Sciences*.

[30] Zhang L.-Z., Meng S.-S., He D.-M. (2016). Three-dimensional measurement and cluster analysis for determining the size ranges of Chinese temporomandibular joint replacement prosthesis. *Medicine*.

[31] He J., Wang M., Lu C. (2003). Relative analysis on the anatomic parameters of condyoid process of mandible and glenoid fossa of TMJ. *Chinese Journal of Conservative Dentistry*.

[32] Li F., Wang Q., Xia F. X., Zhang M. (2000). TMJ form comparison among subjects with different occasions: an MRI survey. *Journal of Dental Research*.

